# Cervical Cancer Correlates with the Differential Expression of Nicotinic Acetylcholine Receptors and Reveals Therapeutic Targets

**DOI:** 10.3390/md17050256

**Published:** 2019-04-28

**Authors:** Yiqiao Liu, Jiang Qian, Zhihua Sun, Dongting Zhangsun, Sulan Luo

**Affiliations:** Key Laboratory of Tropical Biological Resources of Ministry of Education, Key Lab for Marine Drugs of Haikou, Hainan University, Haikou 570228, Hainan, China; yiqiaoliu94@163.com (Y.L.); veilness@163.com (J.Q.); zhihuasun918@163.com (Z.S.); zhangsundt@163.com (D.Z.)

**Keywords:** cervical cancer, nicotinic acetylcholine receptors, differential expression, cell proliferation, α*-conotoxins

## Abstract

Nicotinic acetylcholine receptors (nAChRs) are associated with various cancers, but the relation between nAChRs and cervical cancer remains unclear. Therefore, this study investigated the differential expression of nAChR subunits in human cervical cancer cell lines (SiHa, HeLa, and CaSki) and in normal ectocervical cell lines (Ect1/E6E7) at mRNA and protein levels. Two specific nAChR subtype blockers, αO-conotoxin GeXIVA and α-conotoxin TxID, were then selected to treat different human cervical cancer cell lines with specific nAChR subtype overexpression. The results showed that α3, α9, α10, and β4 nAChR subunits were overexpressed in SiHa cells compared with that in normal cells. α9 and α10 nAChR subunits were overexpressed in CaSki cells. α*-conotoxins that targeted either α9α10 or α3β4 nAChR were able to significantly inhibit cervical cancer cell proliferation. These findings may provide a basis for new targets for cervical cancer targeted therapy.

## 1. Introduction

Cervical cancer is one of the most common female malignancies in the world with 570,000 cases and 311,000 deaths globally in 2018 [1]. It poses a serious threat to women’s health, especially in less developed countries [2]. Persistent infection of high-risk human papillomavirus (HPV) is a major risk factor for cervical cancer [3,4]. Although HPV vaccines are able to effectively prevent infection, they cannot cure the identified infections [5]. The clinical treatments of cervical cancer are now mainly based on surgical treatment, supplemented with chemotherapy and radiation therapy, which are usually accompanied by severe systemic toxicity and chemical resistance. With the continuous development of molecular biology, targeted therapy has attracted wide attention due to its high efficacy, specificity, and low adverse reactions. It has been applied to some tumors and produced a marked effect. However, the targeted therapy of cervical cancer is still in the basic research period, and finding an effective target is of great importance.

Nicotine acetylcholine receptors (nAChRs) are ligand-gated ion channels composed of five different types of subunits, including α, β, δ, ε, or γ, wherein α and β are the main subunits. Initially, it was regarded that nAChRs existed only in the nervous system. Now there is growing evidence showing that nAChRs are also present in non-neuronal cells and tissues, which suggests that nAChRs may play a role in other biological processes besides synaptic transmission [6,7]. As research on nAChRs function progresses, the close relationship between nAChRs and tumor development is gradually understood. Various studies have described nAChRs as key molecules that are involved in tumor development such as tumor growth, angiogenesis, metastasis, and apoptosis [8,9,10]. The regulation of nAChRs is considered to be a promising part of anticancer therapy.

Previous studies have shown that the expression of nAChRs is associated with a variety of cancers. For example, α7 nAChR is in connection with bladder cancer [11], colon cancer [12], gastric cancer [13], head and neck squamous cell carcinoma [14], and pancreatic cancer [15]. In lung cancer, the expression of α7 nAChR is related to nicotine-mediated proliferation, pro-angiogenesis, and metastasis, which can be eliminated by α7 nAChR-specific inhibitors [16,17,18]. It was also found that nAChR genes CHRNA3, CHRNA5, and CHRNB4 were necessary for the survival of small cell lung cancer (SCLC) and that α-conotoxin AuIB, a selective antagonist of α3β4 nAChR, inhibited the survival of SCLC cells [19,20]. Moreover, reduction of α9-nAChR expression in human breast cancer cells by RNA interference inhibited the growth of tumors [21]. Shih-Hsin Tu et al. also found that downregulation of α9-nAChR expression significantly reduced nicotine-induced proliferation of breast cancer cells [22]. However, the understanding of nAChRs in cervical cancer is limited.

In this study, the differential expression of nAChRs in human cervical cancer and human normal ectocervical cell lines was investigated to determine which nAChR subunits may be potential targets for cervical cancer. α*-conotoxins, the selective antagonists of nAChRs, may be the potential new marine drugs for cervical cancer targeted therapy.

## 2. Results

### 2.1. Qualitative Analysis of nAChR Subunits Expression in Human Cervical Cancer by PCR

**Figure 1:**
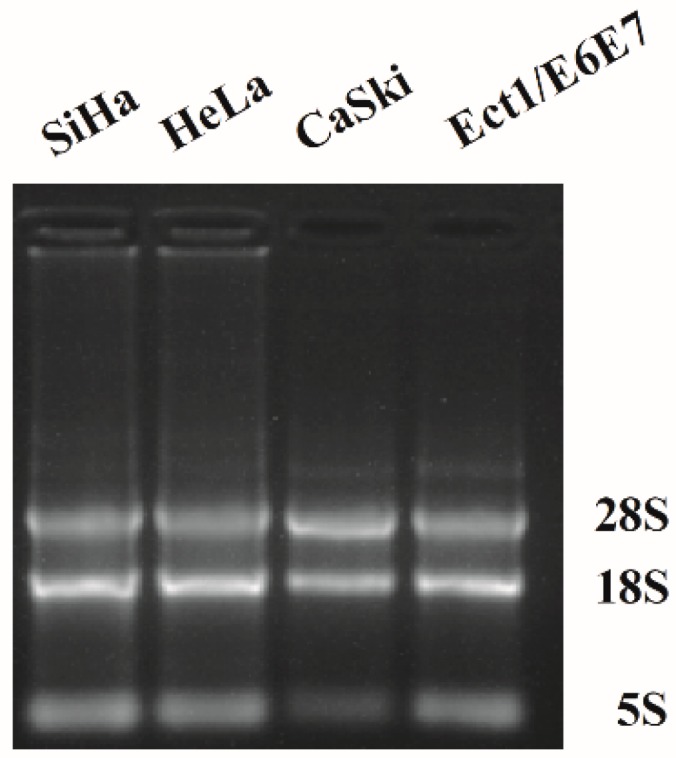
Gel electrophoresis of PCR products. Clear 28S, 18S, and 5S rRNA bands showed integrity of the RNA in human cervical cancer cell lines of SiHa, HeLa, CaSki, and human ectocervical cancer cell line of Ect1/E6E7.

PCR amplification was performed to detect whether nAChR subunits were expressed in human cervical cancer cell lines and the human normal ectocervical cell line. A total of RNAs were extracted from the collected SiHa, HeLa, CaSki, and Ect1/E6E7 cells. After a 1% formaldehyde degrading agarose gel test, 28S, 18S, and 5S bands were observed, which indicated that RNA had good integrity (Figure 1). RNA concentrations and OD260/280 as well as OD260/230 ratios were measured with the NanoDrop 2000 spectrophotometer (Thermo Scientific, Waltham, MA, USA). The latter two were used to evaluate the purity of the samples. The 260/280 ratio and 260/230 ratio of each cell were about 2, showing that the extracted RNA was of good purity with a low content of polyphenols and carbohydrates. The cDNA synthesized by reverse transcription of RNA was then used as a template to amplify 13 nAChR subunits using primers directed against the LBD region (α1-α7, α9, α10, and β1-β4).

**Figure 2:**
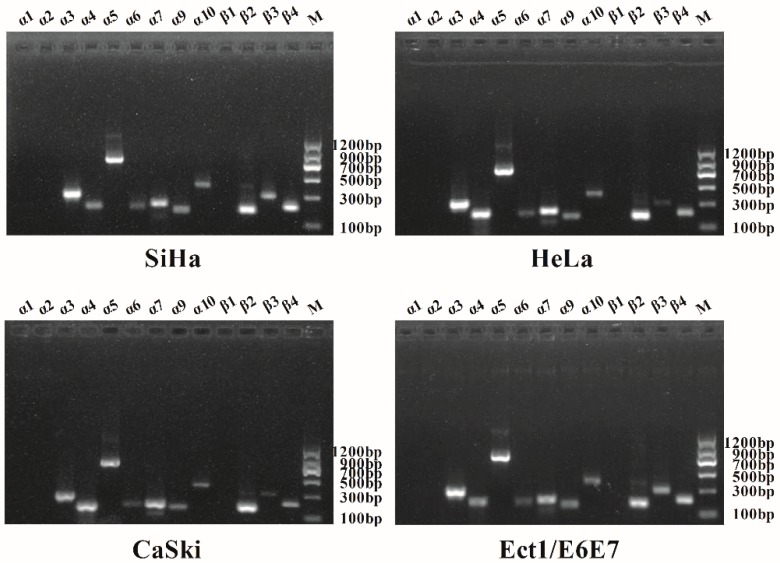
Detection of each nAChR subunit mRNA presented in the human cervical cancer cell lines (SiHa, HeLa, and CaSki) and the human normal ectocervical cell line (Ect1/E6E7) by PCR. The PCR products were amplified with specific primers for α1-α7, α9, α10, and β1-β4 nAChR subunits. The presence of α3-α7, α9, α10, and β2-β4 nAChR was indicated in all four cell lines. The correctness of the obtained PCR products was confirmed by sequencing. No mRNA expression of α1, α2, and β1 nAChR subunits was detected under our experimental conditions.

Gel electrophoresis results displayed the expected amplification bands of α3-α7, α9, α10, and β2-β4 nAChR subunits. No bands of α1, α2, and β1 nAChR subunits were detected under our experimental conditions. Subsequently, the sequencing results showed that the sequence was completely correct, which further verified the existence of these subunits. With all this information, it can be concluded that α3-α7, α9, α10, and β2-β4 nAChR subunits were expressed in our tested cells (Figure 2).

### 2.2. Quantitative PCR Detection of nAChR Subunits Expression

**Figure 3:**
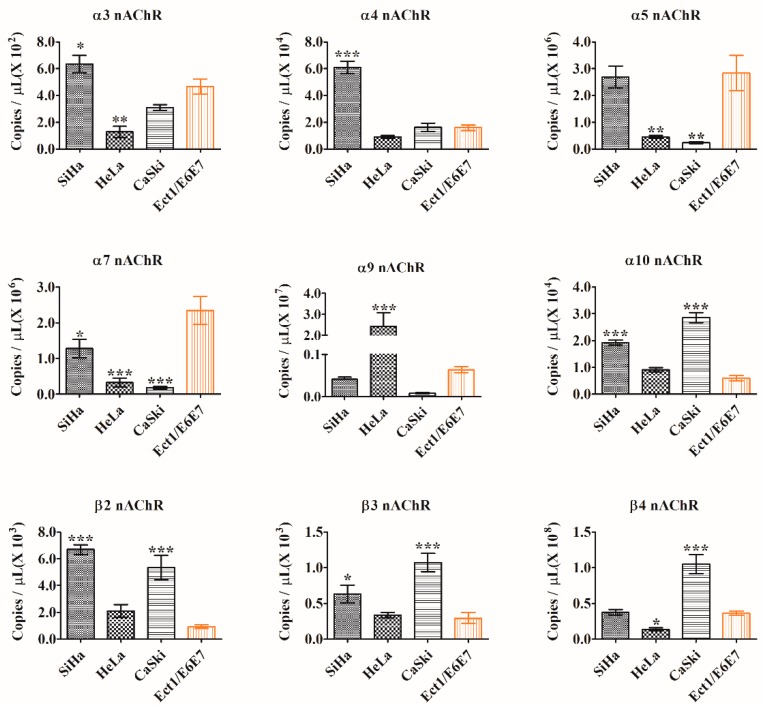
mRNA expression levels of different nAChR subunits in human cervical cancer cell lines (SiHa, HeLa, and CaSki) and the human normal ectocervical cell line (Ect1/E6E7). Initial copies of three human cervical cancer cell lines were compared with that of the human normal ectocervical cell line. Data were shown as means ± SEM of three experiments. * *p* < 0.05, ** *p* < 0.01, and *** *p* < 0.001 versus control cell Ect1/E6E7.

qPCR was conducted to quantitatively estimate the existing nAChR subunits expression at the mRNA level. The standard curve equation of each gene was shown in Table 1, and all the correlation coefficients R^2^ were greater than 0.99, indicating a good linear relationship within the diluted concentration range. Initial copies of nAChR subunits mRNA expression in cells were demonstrated in Table 2. Figure 3 manifested the expression of nAChR subunits in SiHa, HeLa, CaSki, and Ect1/E6E7 cells. The expression differences between the three cervical cancer cells and one normal ectocervical cell were compared. The normal ectocervical cell line Ect1/E6E7 was used as control.

α3 nAChR subunit was highly expressed in SiHa cells (*p* = 0.0402), and the average initial copies were 1.36 times larger than those of normal cell line Ect1/E6E7. In the HeLa cell line, whose average initial copies was about 27% of those of normal cells, the α3 nAChR subunit was poorly expressed (*p* = 0.0012). In CaSki cells, its expression was not significantly different from that in normal cells.

The expression of α4 nAChR subunit showed no significant difference in either HeLa or CaSki cells compared with that in normal cells. However, in SiHa cells, the average initial copies were 3.81 times as many as those in normal cells, which indicated the overexpression of the α4 nAChR subunit (*p* = 5.6 × 10^−6^).

For the α5 nAChR subunit, its expression in SiHa cells was similar to that in normal cells, while its expression in HeLa and CaSki cells was lower (*p* = 0.0024, *p* = 0.0014, respectively). The average initial copies in these two cell lines were about 16% and 8% of those in normal cells, respectively.

In terms of the expression of the α7 nAChR subunit, poor expression was observed in SiHa, HeLa, and CaSki cells (*p* = 0.0146, *p* = 0.0004, *p* = 0.0002, respectively). Specifically, the expression level was CaSki < HeLa < SiHa, and the average initial copies were almost 8%, 14%, and 54% of those in normal cells, respectively.

The expression of the α9 nAChR subunit only demonstrated a difference in HeLa cells (*p* = 0.0009). The average initial copies were 37.64 times larger than those of normal cells. In SiHa and CaSki cells, the expression level was like that in normal cells.

The α10 nAChR subunit was highly expressed in SiHa and CaSki cells (*p* = 5.47 × 10^−5^, *p* = 1.04 × 10^−6^), and the average initial copies were 3.22 and 4.80 times the quantity of the normal cells correspondingly. No significant difference was found in HeLa cells. The expression of β2 nAChR subunit had a likeness to that of the α10 nAChR subunit, namely, it was highly expressed in both SiHa and CaSki cells (*p* = 6.99 × 10^−5^, *p* = 0.0004, respectively) with the average initial copies being 7.19 and 5.75 times larger than those of normal cells, respectively. The same trend also appeared in the expression of the β3 nAChR subunit; it was overexpressed in SiHa and CaSki cells (*p* = 0.0425, *p* = 0.0006, respectively), and the average initial copies were 2.15 and 3.63 times as many as those in normal cells, respectively.

For the β4 nAChR subunit, the expression in SiHa cells was not significantly different from that in normal cells. However, in HeLa cells, the average initial copies were almost 37% of those in normal cells (*p* = 0.0488), and they showed a lower expression than normal cells. On the contrary, in CaSki cells, the expression of the β4 nAChR subunit was highly expressed, and the average initial copies were 2.88 times larger than those in normal cells (*p* = 0.0001).

### 2.3. Detection of nAChR Subunits by Western Blot

**Figure 4:**
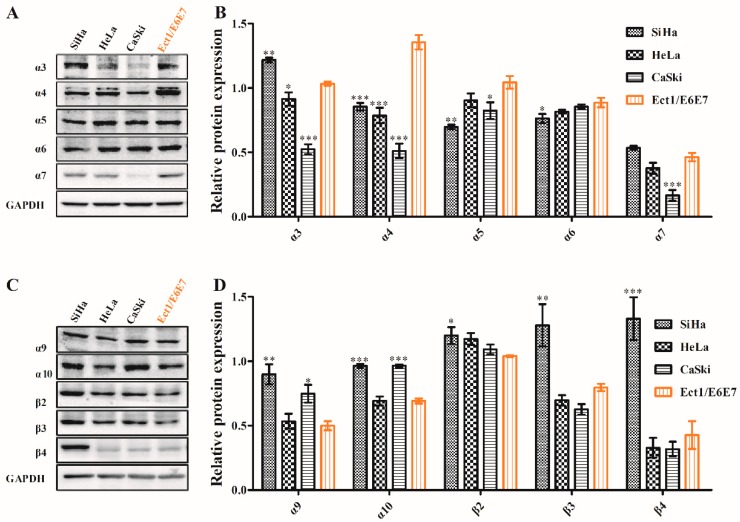
Western blot analysis of nAChR subunit expression in human cervical cancer cell lines (SiHa, HeLa, and CaSki) compared with the human normal ectocervical cell line (Ect1/E6E7). (**A**,**C**) Western blot images of α3, α4, α5, α6, α7, α9, α10, β2, β3, and β4 nAChR. (**B**,**D**) Quantification analysis of western blot for α3, α4, α5, α6, α7, α9, α10, β2, β3, and β4 nAChR subunits. GAPDH was used as the protein loading control. Protein expression levels (relative to GAPDH) were determined. Data were shown as means ± SEM of three experiments. * *p* < 0.05, ** *p* < 0.01, and *** *p* < 0.001 versus control cell Ect1/E6E7.

As shown in Figure 4, all cervical cell lines clearly showed expression of nAChRs when Western blot analysis was performed. In the same way, comparisons between human cervical cancer cell lines and the human normal ectocervical cell line indicated that there were significant differences between their expression of nAChR subunits.

The α3 nAChR subunit was highly expressed in SiHa cells (*p* = 0.0053), and its protein expression level was 1.18 times higher than that of normal cells. Its expression in HeLa and CaSki cells, instead, was low (*p* = 0.0421, *p* = 6.42 × 10^−6^, respectively) and was lowest in CaSki cells. The protein expression level in CaSki cells was 51% of that of normal cells.

Compared with the normal cell line Ect1/E6E7, the expression of the α4 nAChR subunit was lower in all three cell lines SiHa, HeLa, and CaSki (*p* = 0.0001, *p* = 5.47 × 10^−5^, *p* = 3.01 × 10^−6^, respectively). The expression level was CaSki < HeLa < SiHa, and their protein expression was 38%, 58%, and 63% of that of normal cells, respectively.

The α5 nAChR subunit was poorly expressed in SiHa and CaSki cells (*p* = 0.0011, *p* = 0.0138, respectively). In SiHa cells, the expression level of the α5 nAChR subunit was the lowest, with the expression level being 67% of that of normal cells. In HeLa cells, no significant differential expression was found.

For α6 nAChR subunit, its lower expression only was shown in SiHa cells (*p* = 0.0142) with the expression level being 86% of that in normal cells. And there was no significant difference of α6 nAChR subunit expression in other two cells when compared with the normal cells.

The protein expression level of α7 nAChR subunit in SiHa and HeLa cells was not significantly different from that in normal cells. However, it was poorly expressed in CaSki cells (*p* = 0.0003), and its protein expression was 36% of that of normal cells.

The α9 nAChR subunit was highly expressed in SiHa and CaSki cells (*p* = 0.0019, *p* = 0.0220, correspondingly), and its protein level was 1.80 and 1.50 times higher than that of normal cells, respectively. However, its expression in HeLa cells was similar to that in normal cells. The expression trend of the α10 nAChR subunit was the same as that of the α9 nAChR subunit. The α10 nAChR subunit was highly expressed in SiHa and CaSki cells (*p* = 2.43 × 10^−5^, *p* = 2.42 × 10^−5^, respectively), and the expression level was almost the same in both cells. They expressed 1.40 times higher than normal cells.

In relation to the expression of the β2 nAChR subunit, it was highly expressed in SiHa cells (*p* = 0.0341), and its protein level was 1.15 times higher than that of normal cells. The expression of HeLa and CaSki cells was not significantly different from that of normal cells. Similar expression trends were found in the β3 and β4 nAChR subunits. A high expression of the β3 nAChR subunit was found only in SiHa cells (*p* = 0.0045), with the expression level being 1.61 times higher than that of normal cells. The β4 nAChR subunit’s expression in SiHa cells was 3.12 times that of normal cells, which meant it was highly expressed in this cell line (*p* = 0.0004).

### 2.4. Effects of α*-Conotoxins on Cancer Cells

The MTT assay was used to evaluate the inhibition of α*-conotoxins on cervical cancer cells. The α9 and α10 nAChR subunits were overexpressed in SiHa and CaSki cells, and the α3 and β4 nAChR subunits were also overexpressed in SiHa cells. Thus, SiHa and Ect1/E6E7 cells were both treated with αO-conotoxin GeXIVA, a selective antagonist of α9α10 nAChR, or TxID, a selective antagonist of α3β4 nAChR. CaSki cells were also treated with GeXIVA. The two peptides were obtained by chemical synthesis and two-step oxidation in our laboratory [23,24,25].

**Figure 5:**
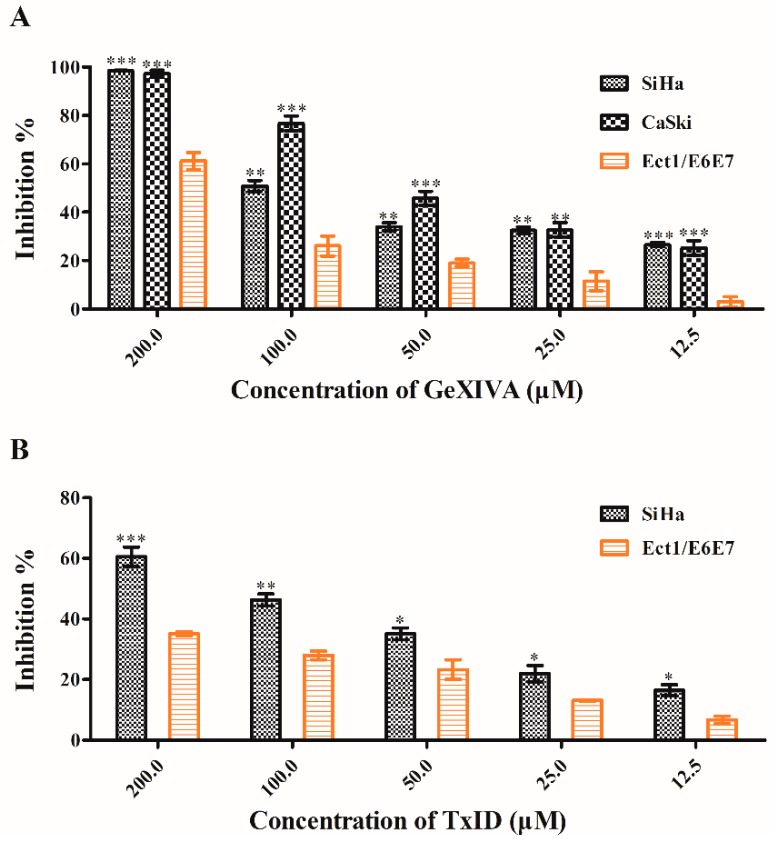
The results of the MTT assay for cell proliferation. Cells were treated with various concentrations of αO-conotoxin GeXIVA or α-conotoxin TxID for 48 h. The inhibition rate of drugs on cancer cells and normal cells at the same concentration was compared. (**A**) Inhibition rate of GeXIVA on SiHa, CaSki, and Ect1/E6E7 cells. (**B**) The inhibition rate of TxID on SiHa and Ect1/E6E7 cells. Data were shown as means ± SEM of three experiments. * *p* < 0.05, ** *p* < 0.01, and *** *p* < 0.001 versus control cell Ect1/E6E7.

In SiHa cells, both GeXIVA and TxID significantly inhibited the proliferation of cancer cells (Figure 5). To be exact, whatever the drug or the concentration was, there were significant differences in the inhibition rates of SiHa cells and Ect1/E6E7 normal cells. The inhibitory effect of GeXIVA is more pronounced than that of TxID.

In CaSki cells, GeXIVA contributed to the inhibition of cell proliferation (Figure 5). Compared with Ect1/E6E7 normal cells treated with the same drug at the same concentration, the inhibition of cancer cells at any concentration of GeXIVA used was significantly higher than that of normal cells.

## 3. Discussion

Recent studies have shown that nAChRs are in close relation with tobacco-related diseases, especially cancer [20,26,27]. Nicotine, the main component of tobacco, can regulate the proliferation, apoptosis, migration, and angiogenesis of cancer cells by activating nAChRs in vivo. In fact, differential expression of nAChRs has been a hot topic of research. It is reported that differential expression of nAChRs is closely related to the development of some cancers, such as the high expression of α7 nAChR in lung cancer and α9 nAChR in breast cancer [21,28]. It can be hypothesized that differential expression of nAChRs might be common in cancers and took three human cervical cancer cell lines as well as one human normal ectocervical cell line as the objects of study.

Previously, Calleja-Macias et al. [29] detected the expression of nAChR in CaSki, SiHa, and HeLa cell lines by RT-PCR and confirmed the successful translation of α5 and β1 corresponding to the two strongest RNA signals in SiHa and HeLa cells by Western blot. Their findings were extended by our study by showing the differential expression of nAChR subunits at the level of mRNA, and protein between human cervical cancer cells and the human normal cell line. However, there were different results in the expression of some nAChR subunits at the level of mRNA and protein (α6 nAChR was not included because the expression of its mRNA was too low to be quantified). Compared with the expression at mRNA level, the expression of α3, α10, β2, and β3 proteins was the same in SiHa and HeLa cells, but the expression of other subunits was different. In CaSki cells, only the expression of α5, α7, and α10 were consistent at the two levels. Though this may seem to be contradictory, the inconsistent expression of a gene at the mRNA and protein levels is not typical but common. In fact, the details of the relation between mRNA and its encoded protein are not fully clear [30]. We believe that the possible reasons for the different expression of the nAChR subunits at two levels include, but are not limited to, 1) mRNA stability (in some cases, mRNA degrades rapidly even if it is expressed, which affects protein expression) and 2) a series of powerful and precise regulatory stages, including transcription, post-transcription, and translation, involved in Eukaryotic gene expression [31,32,33], which is highly complex. Ultimately, we decided to focus on protein expression, because it is closer to the functional receptor level than mRNA levels.

As we know, the nicotinic acetylcholine receptors (nAChRs) are transmembrane pentamers composed of five subunits (α, β, γ, δ, and ε), which can be divided into muscle-type nAChR and neuronal-type nAChR. The structure of muscle-type nAChR is fixed and mainly consists of five subunits (2α1, β1, δ, and γ/ε). Neuronal-type nAChR aggregates into different pentamers in the form of homomers (e.g., α7 and α9) or heteromers (e.g., α4β2, α3β4, and α9α10). The nAChR subtypes that are formed by different subunits are different in function and mediate different physiological processes. For example, α4β2-nAChR is associated with nicotine self-administration, reward and dependence, Alzheimer’s disease, and epilepsy [34], and α3β4 nAChR is connected to lung cancer [19,20]. In our study of Western blot, it was found that the expression of α3, α9, α10, β2, β3, and β4 in SiHa cells was upregulated, which seemed to indicate that α3β2 nAChR, α3β4 nAChR, and α9α10 nAChR were dominant. Furthermore, α9α10 nAChR was abundant in CaSki cells.

Several studies show that specific blockers targeting overexpressed nAChRs in human cancer cells have been applied to new cancer treatment strategies. Specific inhibition of α9 nAChR expression by small interfering RNA or compounds extracted from plants can simultaneously inhibit cancer cell growth, soft agar colony formation, and tumor growth in severe combined immune-deficiency (SCID) mice [22,35,36]. Overexpression of α5 nAChR subunit in non-small cell lung cancer (NSCLC) is associated with proliferation, migration, and invasion of cancer cells through ERK1/2 and PI3K/Akt signaling pathways, and silencing this subunit can inhibit the progress of nicotine-related NSCLC [37,38,39]. α7 nAChR is also associated with the tumor progression such as cell growth and death in NSCLC. The selective antagonist of α7 nAChR can inhibit the proliferation, angiogenesis, and metastasis of nicotine in human NSCLC, which is considered as a potential anticancer agent [10,16,40,41,42,43]. Therefore, cells were treated with the specific blocker α*-conotoxins corresponding to different subtypes of nAChRs to determine the role of these highly expressed nAChRs in cervical cancer and the potential therapeutic effect of α*-conotoxins on cervical cancer according to the expression of nAChRs in this study. The therapeutic potential of specific blockers targeting nAChRs is reflected in their ability to selectively act on the definite nAChRs. Previous studies in our lab found that αO-conotoxin GeXIVA could effectively block α9α10 nAChR, while the effect on other nAChR subtypes was little or nothing [23]. Another research in our lab pointed out that α-conotoxin TxID was the potent α3β4 nAChR antagonist, which was 60-fold more active than another antagonist α-conotoxin AuIB [44] that also targeted the α3β4 nAChR [24]. Thus, SiHa cells were treated with GeXIVA or TxID, and CaSki cells were treated with GeXIVA to evaluate their inhibition effects, which may make further explanation of the role of these highly expressed α3, α9, α10, and β4 nAChR subunits in cervical cancer and indicate the putative treatment of α*-conotoxins on cervical cancer due to their high selectivities to α9α10 nAChR and α3β4 nAChR. Results showed that GeXIVA or TxID markedly inhibited the proliferation of SiHa cells. Although it also inhibited the normal cells, the effect was significantly lower than that on cancer cells. In CaSki cells, GeXIVA revealed an obvious inhibition of proliferation, and its inhibitory effect on normal cells was also significantly less potent than that on cancer cells. Given these findings, α9α10 nAChR and α3β4 nAChR may be therapeutic targets for cervical cancer. Furthermore, α*-conotoxins from marine cone snails that inhibit α9α10 or α3β4 nAChRs may provide new clues for cervical cancer targeted therapy.

## 4. Materials and Methods

### 4.1. Cell Lines and Culture Conditions

Three human cervical cancer cell lines (SiHa, HeLa, and CaSki) and one human normal ectocervical cell line (Ect1/E6E7) were studied. SiHa, HeLa, and CaSki have been widely used as model systems for cervical cancer cell lines for years [29]. The Ect1/E6E7 cell line is often used as a normal control in the researches of cervical cancer, as it is very similar to the characteristics of their tissue of origin and primary cells [45,46]. Details of the cells are shown in Table 3. All these cells were obtained from the American Type Culture Collection (ATCC, Manassas, VA, USA). SiHa, HeLa, and Ect1/E6E7 cells were cultured in complete Dulbecco’s Modified Eagle (DMEM, Sangon Biotech, Shanghai, China) supplemented with 10% fetal bovine serum (FBS, Gibco, Grand Island, NY, USA). CaSki cells were cultured in RPMI 1640 (Gibco, Grand Island, NY, USA) containing 10% fetal bovine serum. All four cells were maintained in a humidified incubator with 5% CO_2_ at 37 ℃.

### 4.2. RNA Extraction and Polymerase Chain Reaction (PCR)

Total RNA was extracted from cell lines using TRIzol reagent (Invitrogen, Carlsbad, CA, USA) according to the manufacturer’s instructions. RNA concentrations and OD260/280 and OD260/230 ratios were measured by a NanoDrop 2000 spectrophotometer (Thermo Scientific, Waltham, MA, USA). After that, RNA samples were reverse-transcribed to cDNA in a 20 μL reaction mix (Thermo Scientific, Waltham, MA, USA). The cDNA was then used for the amplification of nAChR subunits α1-α7, α9, α10, and β1-β4. Sequences of the PCR primers (Sangon Biotech, Shanghai, China) are presented in Table 4. The following PCR conditions were used: pre-incubation for 5 min at 95 °C; 35 cycles of denaturation for 30 s at 95 °C, annealing for 30 s at 60 °C, extension for 30 s at 72 °C; and a final extension for 7 min at 72 °C. PCR products were analyzed on a 1.5% agarose gel and then purified from the agarose gel by the Gel Extraction Kit (Omega, Doraville, GA, USA). The products were connected into pMD18-T vector and then used to transform *E. coli.* DH5α (Sangon Biotech, Shanghai, China). Positive clones were screened on Luria-Bertani (LB) plates containing Amp (50 μg/mL). After identification by bacterial liquid PCR, the bacterial liquid was sent to Sangon Biotech (Shanghai, China) for sequencing. Sequencing results were analyzed at the National Center for Biotechnology Information (NCBI, Bethesda, MD, USA).

### 4.3. Quantitative Real-Time Polymerase Chain Reaction (qPCR)

qPCR was performed to better understand the mRNA expression levels of nAChR subunits. Total RNA was isolated using Trizol reagent (Gaithersburg, MD, USA). Four micrograms of total RNA was reverse-transcribed into cDNA using the High Capacity cDNA Reverse Transcription Kit (Thermo Scientific, Waltham, MA, USA) according to the manufacturer’s instructions. SYBR Green Master Mix (Roche, Basel, Switzerland) was used for amplification. The sequences of the qPCR primers (Sangon Biotech, Shanghai, China) were shown in Table 4. The qPCR parameters were 95 °C for 5 min, followed by 40 cycles of 95 °C for 10 s, 60 °C for 30 s, and 72 °C for 30 s. Standard curves for quantifying the initial copies of the target gene were established using plasmids extracted from the bacterial liquid which was confirmed by sequencing. The plasmids were serially diluted with a gradient of 10 times. Standard curves were established using Log ^Copies^ as the abscissa and cycle threshold (CT) as the ordinate. For quantification, the initial copies of the target gene were extrapolated from the standard curve equation. Results were analyzed using qTOWER^3^ G (Analytik Jena, Jena, Germany), and nAChR subunits expression levels in cervical cancer cells were compared with those in human normal ectocervical cells. All tests were performed in triplicate.

### 4.4. Protein Extraction and Western Blotting Assay

Cells were lysed with RIPA lysis buffer (Sangon Biotech, Shanghai, China) on ice and the total proteins were harvested. The concentration of the total proteins was determined by the BCA protein assay kit (Sangon Biotech, Shanghai, China). Proteins were quantified and loading buffer (5×) was added to the proteins, which were boiled for 5 min. Subsequently, equal proteins were separated on a 10% SDS-PAGE gel and transferred to polyvinyl dene fluoride (PVDF) membranes (Merck Millpore, Billerica, MA, USA) that were blocked with 5% non-fat milk at room temperature for 1 h. After that, blocked membranes were incubated with primary antibodies made up in Tris Buffered Saline with Tween (TBST) overnight at 4 °C. The next day, the membranes were washed three times with 1X TBST and were then incubated with secondary antibodies prepared in 5% non-fat milk for 1 h at room temperature. After another three washes, protein bands were visualized by an ECL detection kit (Boster, Wuhan, China) and quantified using ImageJ software. GAPDH was used as a control protein. Antibodies used were as follows: anti-CHRNA3 picoband antibody (A01981-1, Boster, Wuhan, China), anti-Nicotinic Acetylcholine Receptor α4 antibody [EPR4563(2)] (ab124832, Abcam, Cambridge, UK), anti-CHRNA5 antibody (A02359-2, Boster, Wuhan, China), anti-CHRNA6 antibody (11388-1-AP, proteintech, Chicago, IL, USA), anti-CHRNA7 antibody (21379-1-AP, proteintech, Chicago, IL, USA), AChRα9 antibody (8E4)(sc-293282, Santa Cruz Biotechnology, Santa Cruz, CA, USA), anti-CHRNA10 antibody (ab234767, Abcam, Cambridge, UK), anti-nicotinic acetylcholine receptor β2 antibody (ab55980, Abcam, Cambridge, UK), anti-CHRNB3 antibody (orb338493, biorbyt, Wuhan, China), and CHRNB4 antibody (22192-1-AP, proteintech, Chicago, IL, USA).

### 4.5. MTT Assay

According to the above experimental results, cells were treated with α*-conotoxin targeting different subtypes of nAChRs. Three groups were set up: experimental, control, and blank control groups. The experimental group was treated with drugs, while the control group was not. Both the experimental group and the control group contained cells. The blank group was supplemented with culture medium and contained no cells.

Generally, cells were seeded into 96-well plates at a density of 4 × 10^4^ cells/mL. Following incubation for 24 h in a humidified incubator with 5% CO_2_ at 37 °C, αO-conotoxin GeXIVA [23] or α-conotoxin TxID [24] was added at a concentration of 200, 100, 50, 25, and 12.5 μM and incubated for 48 h. Three parallel wells were set in each group. Subsequently, 10 μL of MTT solution (5 mg/mL) was added to each well and incubated for 4 h. The supernatant was removed and a 100 μL solubilization solution was added to each well to dissolve the crystals. After that, the 96-well plates were put on the shaker and shaken for 10 min at low speed to make the crystals fully dissolved. Results were measured using SpectraMax M2 (Molecular Devices, Eugene, OR, USA) at an absorbance of 570 nm. The formula for calculating the inhibition is (1 − (OD_experimental_ − OD_blank control_)/(OD_control_ − OD_blank control_)) × 100%.

### 4.6. Statistical Analysis

All statistical analyses were performed using SPSS 19.0 software. Data are presented as the means ± SEM (n = 3). Significant differences in data (means ± SEM) among different groups were compared using the one-way analysis of variance (ANOVA). For all experiments, results were considered with a statistical significant at *p* < 0.05.

## Figures and Tables

**Table 1:** Standard curves of nAChR subunits applied to quantification.

nAChR Subunits	Regression Equation of Standard Curve	R^2^	CT Range
α3	y = −3.087x + 33.55	0.997	8.18–30.05
α4	y = −3.207x + 39.08	0.995	10.63–29.56
α5	y = −3.042x + 40.70	0.995	8.69–26.07
α7	y = −3.3594x + 42.339	0.991	13.09–29.26
α9	y = −3.058x + 45.17	0.991	12.24–31.01
α10	y = −3.2786x + 33.164	0.999	8.15–28.20
β2	y = −3.304x + 35.73	0.992	10.91–29.61
β3	y = −3.036x + 33.70	0.998	8.74–27.24
β4	y = −3.358x + 48.09	0.996	10.15–26.78

R = correlation coefficient of standard curves. CT = Cycle Threshold. Y = CT value. X = *Log ^(initial copis)^.*

**Table 2:** Initial copies of each nAChR subunit mRNA expression in different cell lines.

nAChR Subunits	SiHa		HeLa		CaSki		Ect1/E6E7 (Control)
	Copies/μL	Significance	Copies/μL	Significance	Copies/μL	Significance	Copies/μL
α3	6.35 × 10^2^	*	1.28 × 10^2^	^##^	3.10 × 10^2^	ns	4.66 × 10^2^
α4	6.09 × 10^4^	***	9.08 × 10^3^	ns	1.63 × 10^4^	ns	1.60 × 10^4^
α5	2.69 × 10^6^	ns	4.55 × 10^5^	^##^	2.37 × 10^5^	^##^	2.84 × 10^6^
α7	1.28 × 10^6^	^#^	3.26 × 10^5^	^###^	1.82 × 10^5^	^###^	2.35 × 10^6^
α9	4.15 × 10^5^	ns	2.42 × 10^7^	***	8.19 × 10^4^	ns	6.43 × 10^5^
α10	1.92 × 10^4^	***	9.14 × 10^3^	ns	2.86 × 10^4^	***	5.96 × 10^3^
β2	6.68 × 10^3^	***	2.10 × 10^3^	ns	5.34 × 10^3^	***	9.29 × 10^2^
β3	6.33 × 10^2^	*	3.37 × 10^2^	ns	1.07 × 10^3^	***	2.95 × 10^2^
β4	3.78 × 10^7^	ns	1.35 × 10^7^	^#^	1.05 × 10^8^	***	3.64 × 10^7^

Data were shown as the average of three experiments. Compared with the control (normal cell line, Ect1/E6E7), *, **, and *** represent upregulation of expression (*p* < 0.05, 0.01, and 0.001, respectively); ^#^, ^##^, and ^###^ represented downregulation of expression (*p* < 0.05, 0.01, and 0.001, respectively); ns represented no significant difference.

**Table 3:** List of the human cervical cancer and human normal ectocervical cell lines.

Cell Lines		Organism	Years	Derivation and Disease
No.	Name			
1	SiHa	Homo sapiens, human	1975	Female, 55 years, Asian, grade II, squamous cell carcinoma
2	HeLa	Homo sapiens, human	1951	Female, 31 years, Black, adenocarcinoma
3	CaSki	Homo sapiens, human	1974	Female, 40 years, Caucasian, epidermoid carcinoma
4	Ect1/E6E7	Homo sapiens, human	1996	Female, 43 years, unknown, normal

**Table 4:** Primers used for PCR and qPCR analysis.

Primer		Accession No.	Sequence	Amplified Region	Length	Application
No	Name					
1	α1 nAChR-F	NM_001039523.2	CGTCTGGTGGCAAAGCT	(7–587) ^a^	581bp	PCR
	α1 nAChR-R		CCGCTCTCCATGAAGTT			
2	α2 nAChR-F	NM_001282455.1	GAGGACCGGCTCTTCAAACA	(1–289) ^a^	289bp	PCR
	α2 nAChR-R		ACTCCCCATCTGCATTGTTGT			
3	α3 nAChR-F	NM_000743.4	CTGGTGAAGGTGGATGAAGTAAA	(109–410) ^a^	302bp	PCR
	α3 nAChR-R		TGGTAATCAAACGGGAAGTAGGT			
4	α3 nAChR-F		AACGTGTCTGACCCAGTCATCAT	(61–199) ^a^	139bp	qPCR
	α3 nAChR-R		AGGGGTTCCATTTCAGCTTGTAG			
5	α4 nAChR-F	L35901.1	ATGAAATTCGGCTCCTGGACCTA	(1–201) ^a^	201bp	PCR, qPCR
	α4 nAChR-R		CGGCAGCCGCCGGATGAC			
6	α5 nAChR-F	BC033639.1	GAGAGGATTATCTGAACCTTCTTCT	(102–869) ^b^	768bp	PCR
	α5 nAChR-R		ACAAGTACTGAAGTGCAGAGACA			
7	α5 nAChR-F		ACTCCACCGGCAAACTACAA	(343–609) ^a^	267bp	qPCR
	α5 nAChR-R		CAGGCGCTTGATTACAAATGA			
8	α6 nAChR-F	NM_004198.3	GGCTGCGTCACATCTGGAA	(155–356) ^a^	202bp	PCR
	α6 nAChR-R		GCTGGTGGAGTCCAGGTTAT			
9	α7 nAChR-F	NM_000746.5	CCACCAACATTTGGCTGCAA	(143–360) ^a^	218bp	PCR, qPCR
	α7 nAChR-R		TATGCCTGGAGGCAGGTACT			
10	α9 nAChR-F	NM_017581.3	TGGCACGATGCCTATCTCAC	(169–340) ^a^	172bp	PCR, qPCR
	α9 nAChR-R		TGATCAGCCCATCATACCGC			
11	α10 nAChR-F	AF199235.2	CTGTTCCGTGACCTCTTT	(4–391) ^a^	388bp	PCR
	α10 nAChR-R		GGAAGGCTGCTACATCCA			
12	α10 nAChR-F		TGACCTCTTTGCCAACTACAC	(12–149) ^a^	138bp	qPCR
	α10 nAChR-R		CACAGATACAGGGTCAGCAC			
13	β1 nAChR-F	NM_000747.2	AGACCTGGAGTGGACTGACT	(159–317) ^a^	159bp	PCR
	β1 nAChR-R		ACGACGCTAATGTCCAGAGC			
14	β2 nAChR-F	NM_000748.2	GGCATGTACGAGGTGTCCTT	(289–473) ^a^	185bp	PCR, qPCR
	β2 nAChR-R		ACCAAGTCGATCTCTGTGCG			
15	β3 nAChR-F	NM_000749.4	GGGTCCGCCCTGTATTACATT	(44–348) ^a^	305bp	PCR
	β3 nAChR-R		GGTCCAGACAACAGTTCCGT			
16	β3 nAChR-F		GGTCCGCCCTGTATTACATTC	(45–190) ^a^	146bp	qPCR
	β3 nAChR-R		AGCGTAACTTGTGGTCTGTC			
17	β4 nAChR-F	U48861.1	CGCTACGAGGGTGTGAACAT	(208–420) ^a^	213bp	PCR, qPCR
	β4 nAChR-R		GTTCTGCTGGTCGAAGGGAA			

^a^ Amplified regions are part of the ligand binding domain (LBD) regions, and the number is counted from the first base of the LBD region; ^b^ the amplified region is the whole LBD region, and the number is counted from the first base of the coding sequence (CDS) region.
